# Refractory Adult Onset Still’s Disease

**DOI:** 10.7759/cureus.1802

**Published:** 2017-10-25

**Authors:** Wahinuddin Sulaiman, Aris Chandran Abdullah, Jerome Tan Tsen Chuen, Shaffie Baba, Norain Karim

**Affiliations:** 1 Medicine, Faculty of Medicine, Universiti Kuala Lumpur Royal College of Medicine Perak; 2 Medicine, Universiti Kuala Lumpur Royal College of Medicine Perak; 3 Medicine, Hospital Raja Permaisuri Bainun, Ipoh; 4 Radiology, Hospital Raja Permaisuri Bainun, Ipoh; 5 Pathology, Hospital Raja Permaisuri Bainun, Ipoh

**Keywords:** pyrexia of unknown origin, adult onset still’s disease, haemophagocytic lymphohistiocytosis, hyperferritinaemia

## Abstract

It is often a challenge and a dilemma for clinicians encountering patients with pyrexia of unknown origin. Numerous tests performed to determine the underlying cause often give inconclusive results. We present a 52-year-old man with undulating fever for more than 10 months with persistent hyperferritinaemia, and negative immunological and serological markers. Despite corticosteroids, disease modifying anti-rheumatic agents and immune-modulator therapy, he succumbed to the illness. A diagnosis of refractory Adult onset Still’s disease complicated by haemophagocytic lymphohistiocytosis was made.

## Introduction

Adult onset Still’s disease (AOSD) is an auto-inflammatory disorder of unknown etiology without definitive diagnostic test and the diagnosis is usually made by exclusion. AOSD may have protean clinical and laboratory manifestations and it may be associated or complicated by haemophagocytic lymphohistiocytosis (HLH) which carries a poor prognosis and high mortality [1], or even mimics other inflammatory joint disorders such as rheumatoid arthritis.

We present a case of pyrexia of unknown origin (PUO) with atypical manifestations of refractory Adult onset Still’s disease based on persistent hyperferritinaemia with possible HLH. The patient fulfilled two major and two minor Yamaguchi criteria for AOSD [2].

## Case presentation

A 52-year-old Malay male presented with a history of undulating fever, temperatures ranging from 36°C to 40°C for more than 10 months. He had myalgia and arthritis over both his ankle joints and with no associated skin rashes. He had no associated respiratory symptoms, such as cough or hemoptysis, and no constitutional symptoms. He had no other comorbidities, and there was no contact with tuberculosis patients nor exposure to livestock or birds. His lifestyle had been very sedentary and he was a heavy smoker.

He was initially seen at another hospital where a diagnosis of an unspecified autoimmune disease was made and treated with hydroxychloroquine 200 mg daily for few months without any improvement.

Clinical examination was unremarkable apart from high temperature and anaemia at presentation. He was neither toxic nor in distress. He was obese and there were signs of arthritis over both his ankles but other joints were not affected. There was no hepatosplenomegaly or lymphadenopathy.

Immunological markers (anti-nuclear antibody, double-stranded deoxyribonucleic antibody, complements level (C3 and C4), rheumatoid factor, anti-citrullinated peptide, extractable nuclear antigens), electrolytes, venereal disease research laboratory (VDRL) test, renal and liver profiles were all normal. Magnetic resonance imaging of the brain revealed multiple lacunar infarcts. His complete blood count at initial stages showed mild anemia with normal platelets and white cell count. However, the acute phase reactants were persistently raised, i.e., serum ferritin in the range of between 3,000 and 7,000 ng/mL (normal: 16-293), erythrocyte sedimentation rate > 100 mm/1st hour, high sensitive C-reactive protein > 100 mg/L (normal < 5) and raised fibrinogen – 7.0 g/L (normal 2–4). Serum triglyceride and D-dimer were elevated at 3.80 mmol/L (normal < 1.71), and 0.8 µg/mL (normal < 0.5), respectively. Septic work-up (blood, urine), hepatitis B and C, HIV screening, blood film for malaria, dengue, chikungunya, mycoplasma and Brucella serology were all negative. IgM for Epstein Barr virus was positive. Tumour markers (alpha-feto protein, cancer antigen 19-9, carcinoembryonic antigen) were also negative. Peripheral blood film showed no evidence of hemophagocytosis or haemolysis. Coombs’ test and antiphospholipid screening were negative. Procalcitonin level was 0.126 ng/mL (normal: <0.5). Echocardiography showed no vegetation. QuantiFERON-TB Gold test (QIAGEN N.V., Venlo, Netherlands) was positive. However, culture for mycobacterium tuberculosis was negative. Positron emission tomography scan revealed increased uptake in major articulation area especially the ankles and foots suggestive of joint inflammation. However, there were no foci of infection identified on contrast study nor was there any other abnormality seen to suggest malignancy. Bone marrow aspiration/trephine biopsy and cerebrospinal fluid (CSF) analysis were inconclusive but no malignant cells were seen. At this juncture, he was diagnosed as adult-onset Still’s disease and was commenced on methotrexate, 10 mg weekly, and prednisolone, 20 mg daily.

**Figure 1:**
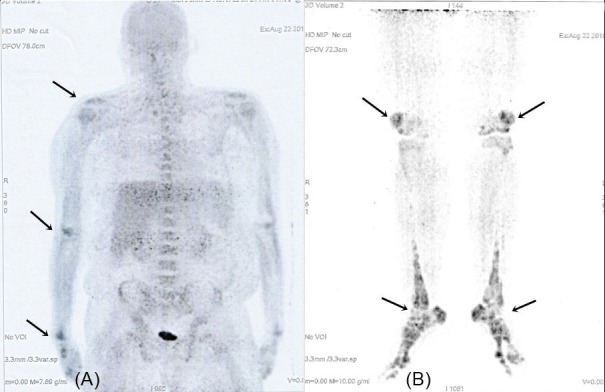
Whole body positron emission tomography scan showed increased uptake in the major articular areas (arrows).

Over the ensuing six months, his condition deteriorated significantly despite treatment with oral prednisolone and methotrexate. As HLH was postulated to be the possible complication, he was commenced on high dose prednisolone 60 mg daily and cyclosporine A 50 mg daily. Unfortunately he developed status epilepticus after three weeks on these medications, eventually requiring ventilatory support. Acute phase reactants were persistently raised and his haemoglobin and platelet counts dropped to as low as 6 g/dl and 20, respectively. He had bleeding tendency and the coagulation profile was mildly deranged, prothrombin time 19.2 sec (normal: 11.0–15.0), international normalized ratio 1.65 (normal: 0.85–1.35). He was treated for coagulopathy with blood products without improvement. There was no evidence of meningism and no source of infection could be identified although white cell count was raised with 80% neutrophils predominance. Electroencephalogram showed diffuse slow wave suggestive of cortical encephalopathy. Computed tomography scan of the brain was normal. A diagnosis of refractory AOSD was made with high possibility of HLH. Intravenous methylprednisolone 500 mg daily followed by intravenous immunoglobulin 5 gm daily for three days were given, with an option to commence plasmapheresis. Interleukin (IL)-6 inhibitor administration was planned pending family approval in view of the cost and availability of therapy. While on ventilatory support his Glasgow Coma Scale showed signs of improvement but there was evidence of organ dysfunction, i.e., renal failure and cardiac arrhythmias (occasional ventricular tachycardia and atrial fibrillation with fast ventricular response). Unfortunately, despite intensive treatment, he succumbed to the illness (Figure 1A, 1B).

## Discussion

PUO can be a great challenge to clinicians globally despite the advancement in medical diagnostic tools. This often results in frequent hospitalization with a repeated battery of investigations. Various underlying etiologies have been implicated, with occult infections being the most frequent cause. Other causes include lymphoproliferative disorders or neoplasms, inflammatory disorders (immune or autoimmune-mediated) and drugs. AOSD has been reported rarely as a cause of PUO [3]. AOSD may present with atypical and protean manifestations or may mimic other inflammatory disorders or chronic infections such as tuberculosis [4]. It may be complicated with HLH which carries a poor prognosis and high mortality, often because of delay in diagnosis and treatment [5]. Although the resources for more specific investigations are limited such as identification of inflammatory cytokines, it may not be able to ascertain the etiology in this patient. Other differentials, such as Q fever, were not considered in this patient based on epidemiological prevalence and incidence in Malaysia. AOSD occurs predominantly in young females (age group 16–35 years) and is rarely reported in the elderly [6].

Refractory AOSD complicated by reactive haemophagocytic lymphohistiocytosis (RHL) has been reported in 12-17% of cases [1, 7]. Early identification of this condition is not an easy task and this can have dire consequences on the health of the patient and even cause death. Hence, prompt treatment with corticosteroids, conventional disease-modifying agents (DMARD), such as methotrexate, immune-modulating agents, immunoglobulin, or even biologic agents, should be considered, and if there is no improvement or worsening is noted, treatment should be escalated promptly. Though the diagnosis is mainly clinical and by exclusion, the patient fulfilled the Yamaguchi criteria, i.e., persistent high fever (>39°C), hepatosplenomegaly, arthritis of ankle joints, and negative anti-nuclear antibody and rheumatoid factor supported by persistent hyperferritinemia as well as exclusion of other possible etiologies. French and Japanese studies have validated the serum ferritin as a diagnostic tool for AOSD when its level reaches five times the upper limit of normal with 80–82% sensitivity and 41–46% specificity, respectively [2]. AOSD has been described as one out of other three conditions, i.e., HLH, antiphospholipid antibody syndrome (APLS), and septic shock in hyperferritinemia syndrome [5]. Although there was minimal definitive clinical and haematological evidence of HLH or RHL, this potentially life-threatening condition was considered very likely due to the presence of hepatosplenomegaly, thrombocytopenia, hypertriglyceridemia, raised fibrinogen index as well as neurological symptoms, i.e., seizure and altered mentation in this patient.

It is now established that refractory cases of AOSD failing conventional therapy may respond to biological agents such as TNF-α, IL-1 and IL-6 inhibitors [8-9]. However, the cost implications of these drugs may limit its availability in less resourceful countries.

## Conclusions

Adult-onset Still’s disease poses a diagnostic challenge to clinicians as it mimics many inflammatory disorders and chronic infections. Up till now, it remains a diagnosis of exclusion, supported by validated sets of diagnostic criteria (Yamaguchi’s criteria 1992 being the most sensitive to date). We highlighted the case of a gentleman with atypical presentation of AOSD complicated by RHL. Despite a stepwise escalation of therapy (‘low dose prednisolone with methotrexate’ followed by ‘high dose prednisolone with cyclosporin’ then ‘intravenous methylprednisolone with intravenous immunoglobulins’), his clinical condition deteriorated accompanied by a persistence of raised inflammatory markers and hyperferritinaemia leading to his demise (multi-organ failure).
